# Multi-Specialty Expert Physician Identification of Extranodal Extension in Computed Tomography Scans of Oropharyngeal Cancer Patients: Prospective Blinded Human Inter-Observer Performance Evaluation

**DOI:** 10.1101/2023.02.25.23286432

**Published:** 2023-02-26

**Authors:** Multidisciplinary OPC ENE Group

## Abstract

**Background:**

Extranodal extension (ENE) is an important adverse prognostic factor in oropharyngeal cancer (OPC) and is often employed in therapeutic decision making. Clinician-based determination of ENE from radiological imaging is a difficult task with high inter-observer variability. However, the role of clinical specialty on the determination of ENE has been unexplored.

**Methods:**

Pre-therapy computed tomography (CT) images for 24 human papillomavirus-positive (HPV+) OPC patients were selected for the analysis; 6 scans were randomly chosen to be duplicated, resulting in a total of 30 scans of which 21 had pathologically-confirmed ENE. 34 expert clinician annotators, comprised of 11 radiologists, 12 surgeons, and 11 radiation oncologists separately evaluated the 30 CT scans for ENE and noted the presence or absence of specific radiographic criteria and confidence in their prediction. Discriminative performance was measured using accuracy, sensitivity, specificity, area under the receiver operating characteristic curve (AUC), and Brier score for each physician. Statistical comparisons of discriminative performance were calculated using Mann Whitney U tests. Significant radiographic factors in correct discrimination of ENE status were determined through a logistic regression analysis. Interobserver agreement was measured using Fleiss’ kappa.

**Results:**

The median accuracy for ENE discrimination across all specialties was 0.57. There were significant differences between radiologists and surgeons for Brier score (0.33 vs. 0.26), radiation oncologists and surgeons for sensitivity (0.48 vs. 0.69), and radiation oncologists and radiologists/surgeons for specificity (0.89 vs. 0.56). There were no significant differences between specialties for accuracy or AUC. Indistinct capsular contour, nodal necrosis, and nodal matting were significant factors in regression analysis. Fleiss’ kappa was less than 0.6 for all the radiographic criteria, regardless of specialty.

**Conclusions:**

Detection of ENE in HPV+OPC patients on CT imaging remains a difficult task with high variability, regardless of clinician specialty. Although some differences do exist between the specialists, they are often minimal. Further research in automated analysis of ENE from radiographic images is likely needed.

## INTRODUCTION

Extranodal extension (ENE), a phenomenon where tumor cells extend beyond the capsule of a lymph node with tumor metastasis, is among the most important adverse prognostic factors in oropharyngeal cancer (OPC), and head and neck squamous cell carcinoma (HNSCC) more broadly ^1^. ENE is often used in clinical decision-making to determine the therapeutic approach for human papillomavirus-positive (HPV+) OPC patients. While there is ambiguity with regard to the impact of clinical/radiographic nodal extension in terms of chemoradiation efficacy, large-scale surgical registry data from the National Cancer Database showed that in >66,000 patients, documented ENE was associated with a >60% decrease in overall survival (Hazzard ratio = 1.63) in patients treated surgically ^2^. Currently, existing treatment paradigms recommend adjuvant chemoradiotherapy if ENE is present ^3^. Alternatively, minimally invasive surgery, e.g., trans-oral robotic surgery, may be preferred if ENE is not present. Therefore, accurate determination of ENE status is crucial for appropriate treatment stratification, which may have significant impacts on patient outcomes.

The current gold-standard approach to identify ENE status in OPC patients involves histopathological evaluation of lymph nodes ^1^. Radiological identification of ENE using commonly available imaging modalities, such as computed tomography (CT), has long been seen as an attractive alternative for non-invasive determination of ENE. Unfortunately, numerous studies have demonstrated that clinician-based radiological identification of ENE in OPC is prone to high variability and poor discriminative performance ^4-8^. Naturally, most of these studies have specifically investigated the discriminative ability of diagnostic radiologists. However, contemporary evaluation and treatment of OPC is typically dependent on the consensus of a multidisciplinary team ^9,10^, with diverse input from clinicians specialized in radiology, surgery, and radiation oncology. Therefore, it is of vital importance to investigate and understand differences between clinical specialties in the interpretation of radiological detectability of ENE, in addition to overall observer performance.

In this study, using a large number of clinician annotators, we prospectively benchmarked specialty-specific discriminative ability of ENE in HPV+ OPC. Through the use of various measures of discriminative performance and observer variability, we probed the underlying relationships between radiologists, surgeons, and radiation oncologists in their interpretation of ENE. Additionally, we prospectively determine the relative intra- and inter-observer performance of expert physicians for detection of extranodal extension using an in silico blinded performance benchmarking task.

## MATERIALS & METHODS

### Clinician annotator characteristics

Thirty-four expert clinician annotators from various medical specialties were recruited for this prospective study - 11 radiologists, 12 surgeons, and 11 radiation oncologists. All observers provided informed consent to have their data utilized in this study.

### Patient and imaging characteristics

Twenty-four patients with a pathologically confirmed diagnosis of HPV+ OPC were included in this analysis. Demographic characteristics of patients used in this study are shown in Table 1. All patients received lymph node dissection confirming the presence or absence of pathological ENE. Specifically, lymph nodes from 17 patients exhibited the presence of histopathological ENE, while lymph nodes from the 7 remaining patients did not (ENE absent). Pre-surgery contrast-enhanced CT images for these patients were retrospectively acquired from The University of Texas MD Anderson Cancer Center picture archiving system. All images were collected in Digital Imaging and Communications in Medicine (DICOM) format. Data were collected under a HIPAA-compliant protocol approved by The University of Texas MD Anderson Cancer Center Institutional Review Board (RCR03-0800 and PA19-0491) which gave ethical approval for this work. CT images were acquired on various scanner devices (GE Discovery CT750 HD = 16; GE Revolution HD = 3; GE LightSpeed VCT = 3; GE Revolution GSI = 1; Siemens SOMATOM Edge Plus = 1) using a diagnostic head and neck CT imaging protocol with intravenous contrast administration. CT acquisition parameters are shown in Table 2.

### Image processing

Patient CT scans were exported as DICOM radiotherapy structure (RTS) files and converted to Neuroimaging Informatics Technology Initiative (NIfTI) format for ease of use using the DICOMRTTool v.3.2.0 Python package ^11^. In order to minimize observer exposure to irrelevant tissue, CT images were cropped to the cephalad border of the sternum and inferior border of the hard palate. In order to measure intraobserver variability, images from a random subset of 6 patients (4 with ENE present, 2 with ENE absent) were added twice in random positions of the final case set, leading to a total of 30 cases: 21 with ENE present and 9 with ENE absent.

### Survey Instrument

Anonymized NIfTI formatted images for the 30 cases were independently shown to the clinician observers using 3D Slicer ^12^ via remote control of the 3D Slicer screen over Zoom. For each patient’s contrast enhanced CT scan, physicians scrolled through the axial, sagittal, and/or coronal planes of the images and answered nine questions (Appendix, A1). Seven commonly applied radiological features ^13^ were evaluated by annotators: indistinct capsular contour, irregular lymph node margin, thick-walled enhancing nodal margin, perinodal fat stranding, perinodal fat plane or gross invasion, nodal necrosis, and nodal matting. Annotators marked “present” or “absent” for each of the features if any of the lymph nodes in the patient met the criteria. Additionally, annotators were asked if ENE was present or absent in any lymph nodes and to provide an estimate of their confidence in predicting ENE status (0-100% certain).

### Discriminative Performance

Discriminative performance was quantified using various evaluation metrics, including accuracy, area under the receiver operating characteristic curve (AUC), sensitivity, and specificity. These metrics were selected due to their ubiquity in literature and relevance to ENE discrimination ^14-16^. Observer predictions were directly used for calculating accuracy, sensitivity, and specificity, while observer confidence was used for calculating AUC. Accuracy, AUC, sensitivity, and specificity were measured from 0 to 1, with higher values being deemed better. The Brier score ^17^ was also investigated to determine the calibration (i.e., reliability of observer confidence) of individual predictions. Brier score values were measured from 0 to 1, with lower values being deemed better. Aggregated metric performance was reported as median values with corresponding interquartile range (IQR) values. Mann-Whitney U tests were used to compare performance metrics between clinical specialties due to non-normal distributions of data. p values less than or equal to 0.05 were considered significant. All discriminative metrics were calculated in Python v.3.8.8 using the scikit-learn v.1.0.2 package ^18^; Mann-Whitney U tests were calculated using the statannotations v.0.4.4 package.

### Radiographic Criteria Analysis

Overall percentages for the presence of each radiographic criteria across all the cases that were correctly identified (ENE correctly identified as present or ENE correctly identified as absent) were stratified by expert specialty and displayed in tabular format. Logistic regression was performed using R version 4.2.2 to determine significant factors in the correct determination of ENE status.

### Performance Variability

Observer variability was evaluated using multiple methods. Agreement on the status of radiographic features among specialties was assessed by Fleiss’ Kappa using the irr v.0.84.1 package in R ^19^. To measure the reliability of the assessment of ENE by physicians, the intraclass correlation coefficient (ICC) was calculated using the pingouin v.0.5.3 package in Python. Finally, the standard error of measurement (SEm) was calculated using the duplicated cases in order to evaluate the interobserver and intraobserver variability in ENE status assessment using the SEofM v.0.1.0 package in R ^20^.

## RESULTS

### Discriminative Performance

**Figure 1.**
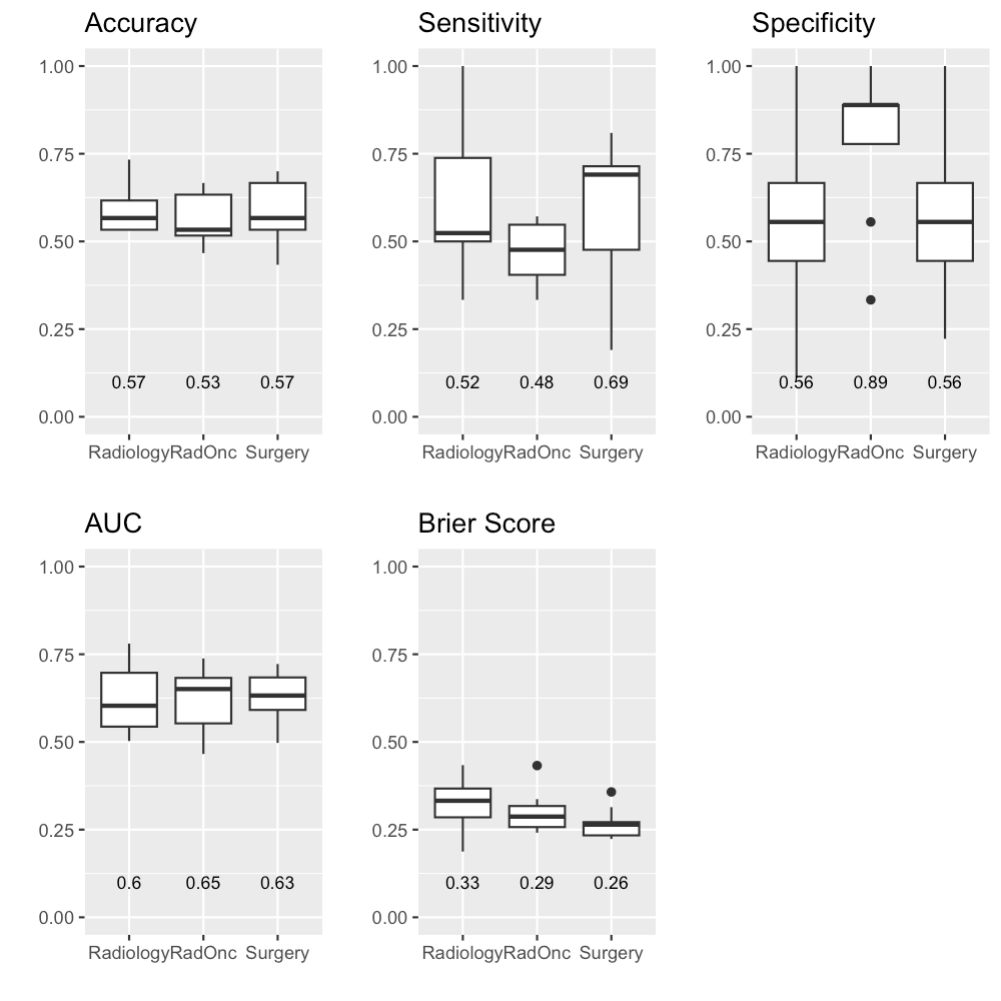
Comparisons of specialty-specific performance in detecting extranodal extension. Accuracy, sensitivity, and specificity, area under the receiver operating characteristic curve (AUC), and Brier scores are shown separately for radiologists (Radiology), radiation oncologists (RadOnc), and surgeons (Surgery). Higher values are deemed superior for all metrics except Brier score (lower = better).

Median (IQR) performance aggregated across specialties was 0.57 (0.10), 0.64 (0.13), 0.28 (0.08), 0.53 (0.27), and 0.61 (0.33) for accuracy, AUC, Brier score sensitivity, and specificity, respectively. Performance metrics aggregated by clinician specialty are shown in Figure 1. Surgeons had the top median scores for accuracy (0.57), Brier score (0.25), and sensitivity (0.69). Radiation oncologists had the top median scores for AUC (0.65) and specificity (0.89). There were significant differences between radiologists and surgeons for Brier score (0.33 vs. 0.26), radiation oncologists and surgeons for sensitivity (0.48 vs. 0.69), and radiation oncologists and radiologists/surgeons for specificity (0.89 vs. 0.56). No other comparisons were significantly different.

### Radiographic Criteria Analysis

The breakdown of annotators' utilization of radiographic criteria in relation to cases that were correctly identified is shown in Table 3. The criteria most observed in aggregate for correct identification of ENE presence was nodal necrosis (92.9%). Similarly, by specialty, nodal necrosis was the most used criteria for radiologists and radiation oncologists in the correct identification of ENE presence (98.6% and 88%, respectively), while irregular lymph node margin was the most used criteria by surgeons (95.3%). Notably, nodal necrosis was also the most observed criteria in aggregate (40.1%) and stratified by specialty (radiologists = 48.2%, radiation oncologists = 40.5%, and surgeons = 32.3%) in the correct identification of ENE absence. The criteria least observed in aggregate (7.1%) and by specialty (radiologists = 3.6%, radiation oncologists = 3.8%, and surgeons = 14.5%) for the correct identification of ENE absence was perinodal fat plane or gross invasion.

Logistic regression was performed to evaluate which radiographic features were associated with the correct prediction of ENE status. As seen in Table 4, identifying the presence of an indistinct capsular contour, nodal necrosis, or nodal matting significantly increased the odds of correctly predicting ENE status. When applying separate regression analyses stratified by specialty, only indistinct capsular contour and perinodal fat plane or gross invasion was significant for radiologists, only nodal necrosis was significant for radiation oncologists, and only nodal necrosis and nodal matting were significant for surgeons.

### Performance Variability

**Figure 2.**
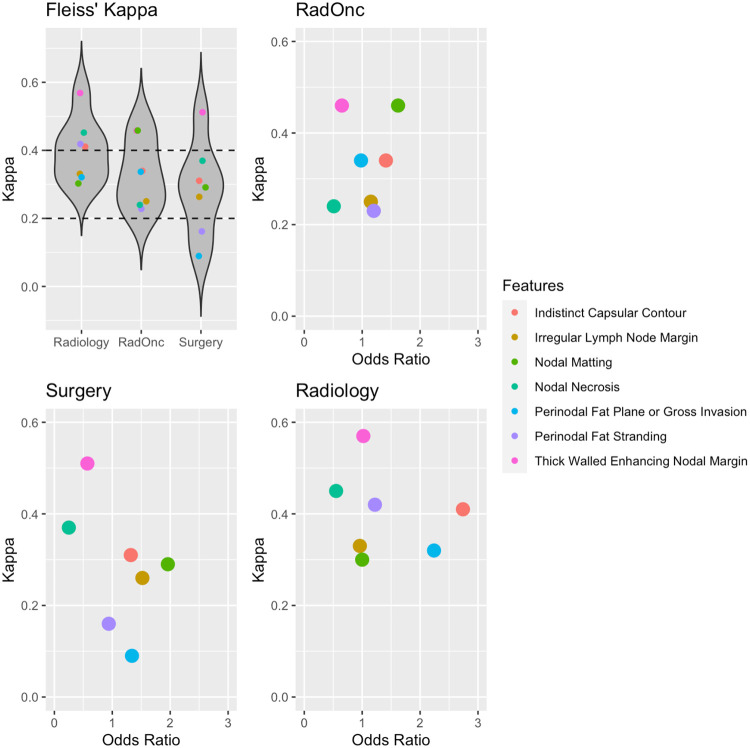
Fleiss’ Kappa for the seven radiographic features for each specialty. Higher values represent greater agreement in the evaluation of presence or absence for each feature. Dotted lines correspond to range of minimal agreement (0.2 < Kappa < 0.4). Subplots show agreement versus odds ratio in correctly determining ENE for each feature stratified by clinician specialty. The top right corner of the subplots represents features with high agreement and high predictive value.

Figure 2 shows Fleiss’ Kappa for the seven radiographic features across specialties. Radiologists had weak agreement (0.4 < Kappa < 0.6) in assessing thick-walled enhancing nodal margin, nodal necrosis, perinodal fat stranding, and indistinct capsular contour, and minimal agreement (0.2 < Kappa < 0.4) in irregular lymph node margin, perinodal fat plane or gross invasion, and nodal matting. Radiation oncologists had weak agreement in nodal matting and thick-walled enhancing nodal margin, and minimal agreement in all other features. Surgeons had weak agreement only in assessing thick-walled enhancing nodal margin, no agreement (0 < Kappa < 0.2) in perinodal fat stranding and perinodal fat plane or gross invasion, and minimal agreement in all other radiographic features.

**Figure 3.**
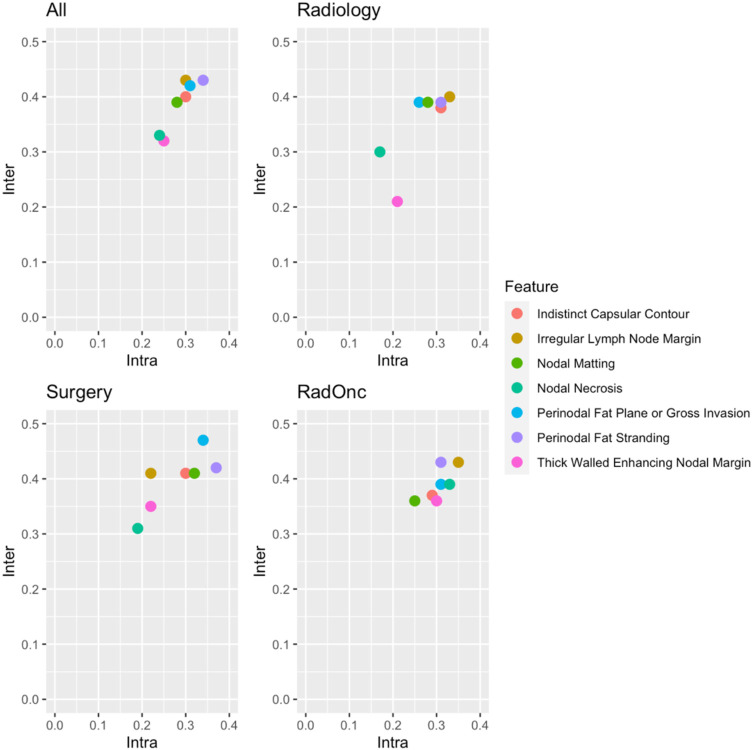
Interobserver vs. intraobserver variability plots as measured with the standard error of measurement. Each colored dot corresponds to a radiographic criterion. Results are presented for all observers and stratified by clinician specialty. Values in the bottom left corner represent features with low interobserver variability and low intraobserver variability, so would be preferred.

To evaluate the intraobserver and interobserver variability in ENE and radiographic feature assessment, the standard error of measurement within each observer and among observers was calculated, respectively. Figure 3 shows that there was generally greater interobserver variability than intraobserver variability. Moreover, surgeons had the highest interobserver variability (perinodal fat plane or gross invasion SEm = 0.47) and highest intraobserver variability (perinodal fat stranding SEm = 0.37). Additionally, as a measure of the consistency in the given results, the ICC between all physicians was calculated using a single rating model resulting in a value of 0.36 (95% confidence interval = [0.26, 51], p-value < 0.0001).

## DISCUSSION

In this study, we queried a large number of clinicians across three different specialties relevant to HPV+ OPC patient management to determine differences in interpretation of radiological ENE. Broadly, we determine that though differences do exist between specialists, they are often minimal. Moreover, due to the difficulty of determining ENE from radiological features, almost all specialists are unanimously poor predictors of ENE status. To our knowledge, this is the largest individual study to investigate radiological interpretation for ENE patients in HNSCC using multiple clinician annotators.

We employed a systematic approach to investigating annotator performance by utilizing several evaluation metrics. A recent meta-analysis reported pooled sensitivity, specificity, and AUC values of 0.77, 0.60, and 0.72, respectively, for CT-based identification of ENE in OPC ^14^. While our aggregated values are notably lower for sensitivity (though still within the 95% confidence interval), our specificity and AUC are similar. Interestingly, when stratified by clinician specialty, radiation oncologists had significantly higher specificity than the other specialties. These results indicate that radiation oncologists are relatively superior at correctly determining the absence of ENE. Specialty-specific factors may have led to an improved ability of radiation oncologists to correctly determine the absence of ENE. Finally, while less commonly investigated, we utilized the Brier score to measure probabilistic prediction accuracy of specialists based on their confidence in their assessment of ENE. We showed that surgeons yielded the lowest (best) Brier score of all specialties and were significantly lower than radiologists, indicating relatively good calibration of predictions, likely due to more conservative estimates of confidence.

In a large-scale meta-analysis for all HNSCC subtypes, it was found that central node necrosis showed high pooled sensitivity, while infiltration of adjacent planes showed a high pooled specificity ^15^. These findings are echoed in our study as nodal necrosis was the most commonly observed feature in aggregate for correctly determining ENE presence, while perinodal fat plane or gross invasion was the least commonly observed feature for correctly determining ENE absence. It should be noted that nodal necrosis was observed in almost all cases correctly identified with ENE and in a large portion of cases correctly identified without ENE, as could be expected for HPV+ OPC ^21^. For surgeons, rather than nodal necrosis, irregular lymph node margin was the most observed criterion for correct identification of ENE presence, which may be linked to their high sensitivity. Notably, on regression analysis, several radiographic criteria were significant contributors to the correct determination of ENE status. Moreover, there were some differences that emerged in significant criteria when stratifying the regression analysis by clinician specialty. However, irregular lymph node margin, thick-walled enhancing nodal margin, and perinodal fat stranding were among the criteria not deemed significant. This is not necessarily surprising given that these criteria have been less routinely reported in ENE studies ^14-16^.

Recent literature in HPV+ OPC ENE identification has suggested that CT radiographic criteria have poor reproducibility among expert observers ^16^, though there could be some improvements in reproducibility when using a high certainty threshold for ENE identification, consolidating operational definitions, and the sharing of experience among observers ^22^. We sought to determine if these findings were consistent when stratified by clinician specialty. Notably, Fleiss’ kappa was always less than 0.6, regardless of specialty or radiographic criteria, consistent with findings from Tran et al. ^16^. As expected, radiographic features that had higher agreement, both overall and within specialties, tended to have lower intraobserver and interobserver variability. Additionally, though there were features with relatively high agreement and low intra/interobserver variability, it is not clear if these features can be used to predict ENE as their presence may not be significantly associated with the correct prediction of ENE, as seen with thick-walled enhancing nodal margin ^15^.

Our study is not without limitations. Firstly, we only investigated a single imaging modality for the identification of ENE status, namely CT. While recent evidence has suggested the incorporation of additional imaging modalities, such as magnetic resonance imaging (MRI) and positron emission tomography (PET), could improve the discrimination of ENE in OPC ^14,23^, CT is among the most ubiquitous imaging modalities available for OPC patients. Therefore, we have chosen to focus on CT as an exemplar imaging modality in this study. Secondly, due to not all patients having complete pathological ground truth information for ENE extent, we did not utilize this as a factor in our analysis. However, it is well known that depending on the ENE extent (i.e., > 2 mm), discriminant capacity often increases ^7^. Finally, while most patients in this dataset only had one positive lymph node, some patients with multiple positive nodes could have added unaccounted for ambiguity in clinician determination of ENE status. Additionally, while pathologic assessment of ENE was used as a gold standard for this study, the accuracy of this assessment method has been questioned in the literature ^24-26^.

Overall, our study reinforces the findings of previous investigations, which caution against relying solely on human interpretation of ENE from radiological imaging. Given the difficulty of ENE detection for human observers regardless of clinical specialty, even when utilizing defined radiographic criteria, it is pertinent that solutions are put forth that could improve or automate this task. In recent years, machine learning approaches have been proposed as accurate and reproducible tools for determining ENE status from radiological images of HNSCC patients ^27-29^. We anticipate these methods to play an increasing role in the clinical utility of radiological determination of OPC ENE status in the future.

## CONCLUSIONS

In summary, by querying 34 clinician annotators across 30 HPV+ OPC cases, we demonstrate that there are minimal differences in CT-based radiologic ENE interpretation between radiologists, radiation oncologists, and surgeons. On average, all annotators performed poorly in discriminating ENE status as determined through various evaluation metrics. Moreover, there was high variability between and within specialties. Future studies should incorporate the utilization of additional complementary imaging modalities (e.g., MRI and PET) and/or automated approaches (e.g., machine learning) that would improve discriminative performance and minimize variability of ENE identification.

## Figures and Tables

**Table 1.** Patient demographic characteristics for the 24 OPC patients used in this study. Values for the first three characteristics are displayed as median and range. All others are displayed the total number of patients.

Demographic Characteristic	Value
Positive lymph nodes (N)	1 (1-7)
Lymph nodes removed (N)	31 (16-47)
Largest lymph node metastasis size (cm)	2.7 (0.6-5)
Sex (N)	-
Male	21
Female	3
Race (N)	-
Non-Hispanic white	20
Hispanic white	3
Black/African American	1
Smoking history (N)	-
Never smoked	18
Previous or current smoker	6
Laterality (N)	-
Right	13
Left	11
T stage (N)	-
1	15
2	9
N stage (N)	-
1	23
2	1
Lymph node levels positive (N)	
2	18
2 and 3	5
3	2
2 and 4	1

**Table 2.** CT image acquisition parameters. Values shown are median (range), except for kilovoltage peak (kVp), which was the same for all patients.

CT Aquisition Parameter	Value
In-plane resolution (mm)	0.49 (0.49 - 0.53)
Slice thickness (mm)	1.25 (1.25 - 1.5)
Exposure time (ms)	1000 (1000 - 1825)
X-ray tube current (mA)	260 (159-409)
kVp (kV)	120

**Table 3.** Use of radiographic criteria in correct identification of extranodal extension (ENE). Values are stratified by cases that were correctly determined as ENE being present or absent and by annotator specialty. For each radiographic criteria, the values shown are the percentage identified as present by observers. Abbreviations: ICC = indistinct capsular contour, ILNM = irregular lymph node margin, TWENM = thick-walled enhancing nodal margin, PFS = perinodal fat stranding, PFPoFI = perinodal fat plane or gross invasion, NN = nodal necrosis, NM = nodal matting.

	ENE correctly identified as present (Patient n = 21 with ENE)							ENE correctly identified as absent (Patient n = 9 without ENE)						
	ICC	ILNM	TWENM	PFS	PFPoFI	NN	NM	ICC	ILNM	TWENM	PFS	PFPoFI	NN	NM
**Radiology**	79.7%	78.3%	82.6%	74.6%	60.1%	98.6%	56.5%	10.7%	7.1%	23.2%	35.7%	3.6%	48.2%	19.6%
**RadOnc**	80.6%	87%	78.7%	61.1%	69.4%	88%	63.9%	15.2%	27.8%	36.7%	13.9%	3.8%	40.5%	15.2%
**Surgery**	87.3%	95.3%	80.7%	48.7%	60.7%	91.3%	67.3%	21%	38.7%	32.3%	19.4%	14.5%	32.3%	22.6%
**All**	82.8%	87.1%	80.8%	61.1%	62.9%	92.9%	62.6%	15.7%	25.4%	31.5%	21.8%	7.1%	40.1%	18.8%

**Table 4.** Logistic regression of correct ENE status prediction using radiographic features and ENE assessment confidence. Bolded p-values indicate significant terms. * = p ≤ 0.05, ** = p ≤ 0.01, *** = p ≤ 0.005.

Term	Coefficient	Standard Error	Odds Ratio	95% CI	P-value
**All Physicians**					
Indistinct Capsular Contour	0.54	0.21	1.71	[1.14, 2.57]	0.01 **
Irregular Lymph Node Margin	0.04	0.20	1.04	[0.7, 1.55]	0.83
Thick-Walled Enhancing Nodal Margin	−0.33	0.18	0.72	[0.51, 1.01]	0.06
Perinodal Fat Stranding	0.09	0.17	1.1	[0.78, 1.54]	0.59
Perinodal Fat Plane or Gross Invasion	0.34	0.20	1.4	[0.94, 2.08]	0.09
Nodal Necrosis	−0.84	0.19	0.43	[0.3, 0.63]	<2e-16 ***
Nodal Matting	0.40	0.17	1.5	[1.06, 2.1]	0.02 *
Assessment Confidence	1.00	0.15	2.73	[2.04, 3.66]	<2e-16 ***
**Radiologists**					
Indistinct Capsular Contour	1.01	0.37	2.74	[1.33, 5.75]	0.01 *
Irregular Lymph Node Margin	−0.04	0.37	0.96	[0.46, 1.96]	0.92
Thick-Walled Enhancing Nodal Margin	0.02	0.32	1.02	[0.54, 1.89]	0.96
Perinodal Fat Stranding	0.20	0.30	1.22	[0.66, 2.20]	0.52
Perinodal Fat Plane or Gross Invasion	0.81	0.38	2.24	[1.07, 4.78]	0.03 *
Nodal Necrosis	−0.59	0.38	0.55	[0.26, 1.16]	0.12
Nodal Matting	0.00	0.31	1.00	[0.54, 1.84]	0.99
Assessment Confidence	0.54	0.26	1.71	[1.03, 2.86]	0.04 *
**Radiation Oncologists**					
Indistinct Capsular Contour	0.34	0.38	2.41	[0.66, 2.96]	0.37
Irregular Lymph Node Margin	0.14	0.35	1.15	[0.57, 2.31]	0.69
Thick-Walled Enhancing Nodal Margin	−0.43	0.31	0.65	[0.35, 1.18]	0.16
Perinodal Fat Stranding	0.18	0.36	1.20	[0.58, 2.43]	0.62
Perinodal Fat Plane or Gross Invasion	−0.02	0.45	0.98	[0.40, 2.34]	0.97
Nodal Necrosis	−0.68	0.32	0.51	[0.27, 0.95]	0.04 *
Nodal Matting	0.48	0.35	1.62	[0.82, 3.19]	0.17
Assessment Confidence	1.33	0.33	3.77	[2.02, 7.38]	<2e-16 ***
**Surgeons**					
Indistinct Capsular Contour	0.28	0.36	1.32	[0.65, 2.67]	0.44
Irregular Lymph Node Margin	0.42	0.38	1.52	[0.71, 3.24]	0.28
Thick-Walled Enhancing Nodal Margin	−0.56	0.30	0.57	[0.31, 1.03]	0.06
Perinodal Fat Stranding	−0.06	0.29	0.94	[0.53, 1.66]	0.83
Perinodal Fat Plane or Gross Invasion	0.29	0.31	1.34	[0.73, 2.43]	0.34
Nodal Necrosis	−1.40	0.36	0.25	[0.12, 0.49]	<2e-16 ***
Nodal Matting	0.67	0.28	1.96	[1.13, 3.41]	0.02 *
Assessment Confidence	1.18	0.26	3.24	[1.98, 5.42]	<2e-16 ***

**Figure A1.**
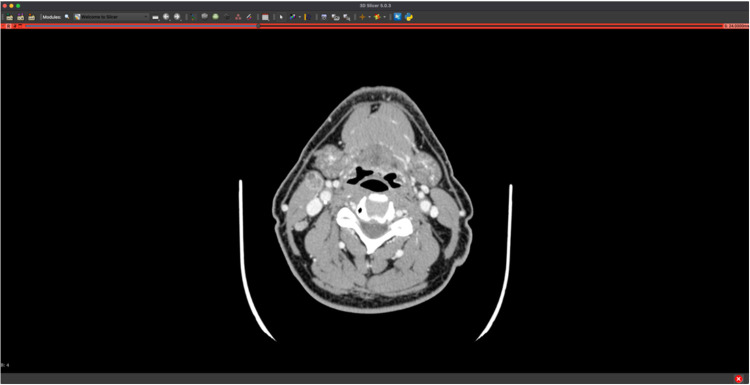

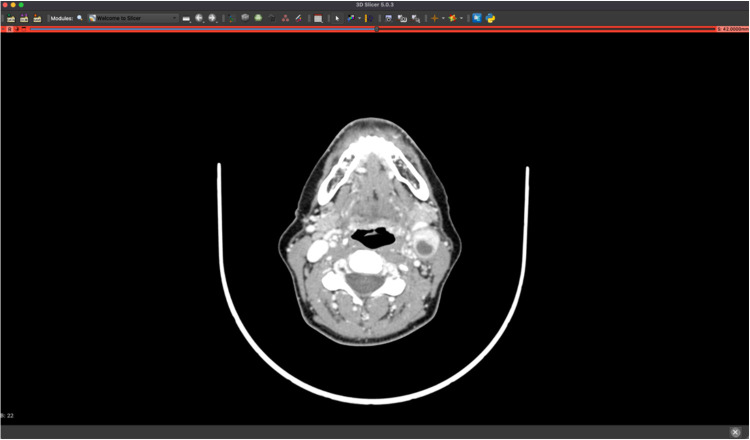
Example of CT scan in 3D Slicer with (top) and without (bottom) ENE presence as seen by observers. Observers could scroll through the scan remotely via Zoom, change planes between axial, sagittal, or coronal, and change the window level and width.

## † Contributing Authors:

Onur Sahin^1^, Kareem A. Wahid ^1^, Nicolette Taku ^1^, Renjie He ^1^, Mohamed A. Naser ^1^, Abdallah S. R. Mohamed ^1^, Antti Mäkitie ^2^, Benjamin H. Kann ^3^, Kimmo Kaski ^4^, Jaakko Sahlsten ^4^, Joel Jaskari ^4^, Moran Amit ^1^, Melissa M. Chen ^1^, Gregory M. Chronowski ^1^, Eduardo M. Diaz Jr. ^1^, Adam S. Garden ^1^, Ryan P. Goepfert ^1^, Jeffrey P. Guenette ^3^, G. Brandon Gunn ^1^, Jussi Hirvonen ^5^, Frank Hoebers ^3^, Nandita Guha-Thakurta ^1^, Jason Johnson ^1^, Diana Kaya ^1^, Shekhar D. Khanpara ^1^, Kristofer Nyman ^2^, Stephen Y. Lai ^1^, Miriam Lango ^1^, Kim O. Learned ^1^, Anna Lee ^1^, Carol M. Lewis ^1^, Anastasios Maniakas ^1^, Amy C. Moreno ^1^, Jeffery N. Myers ^1^, Jack Phan ^1^, Kristen B. Pytynia ^1^, David I. Rosenthal ^1^, Vlad Sandulache ^6^, Dawid Schellingerhout ^1^, Shalin J. Shah ^1^, Andrew G. Sikora ^1^, Max Wintermark ^1^, Clifton D. Fuller ^1^*

^1^ The University of Texas MD Anderson Cancer Center, Houston, USA

^2^ University of Helsinki and Helsinki University Hospital, Helsinki, Finland

^3^ Harvard Medical School, Boston, USA

^4^ Aalto University, Espoo, Finland

^5^ Tampere University, Faculty of Medicine and Health Technology and Tampere University Hospital, Tampere, Finland

^6^ Baylor College of Medicine, Houston, USA
