# Supplementary material for: International Multi-Specialty Expert Physician Preoperative Identification of Extranodal Extension n Oropharyngeal Cancer Patients using Computed Tomography: Prospective Blinded Human Inter-Observer Performance Evaluation
Source: medRxiv. 2024 Jun 8:2023.02.25.23286432. Originally published 2023 Feb 26. Preprint. [Version 2] doi: 10.1101/2023.02.25.23286432 (PMC9980252; doi:10.1101/2023.02.25.23286432)
Supplement: Supplement 1 [file NIHPP2023.02.25.23286432v2-supplement-1.pdf]

medRxiv preprint doi: https://doi.org/10.1101/2023.02.25.23286432; this version posted June 8, 2024. The copyright holder for this preprint (which was not certified by peer review) is the author/funder, who has granted medRxiv a license to display the preprint in perpetuity.
It is made available under a CC-BY 4.0 International license .

Supplementary Material

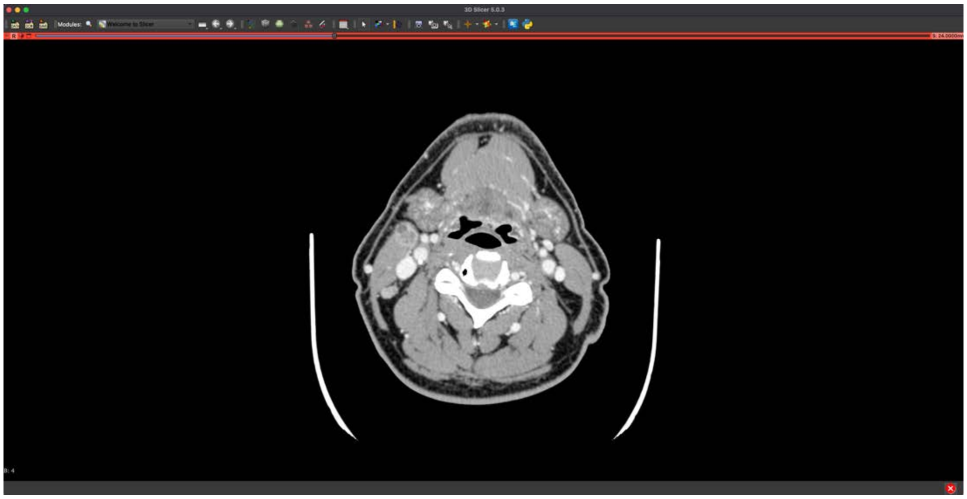

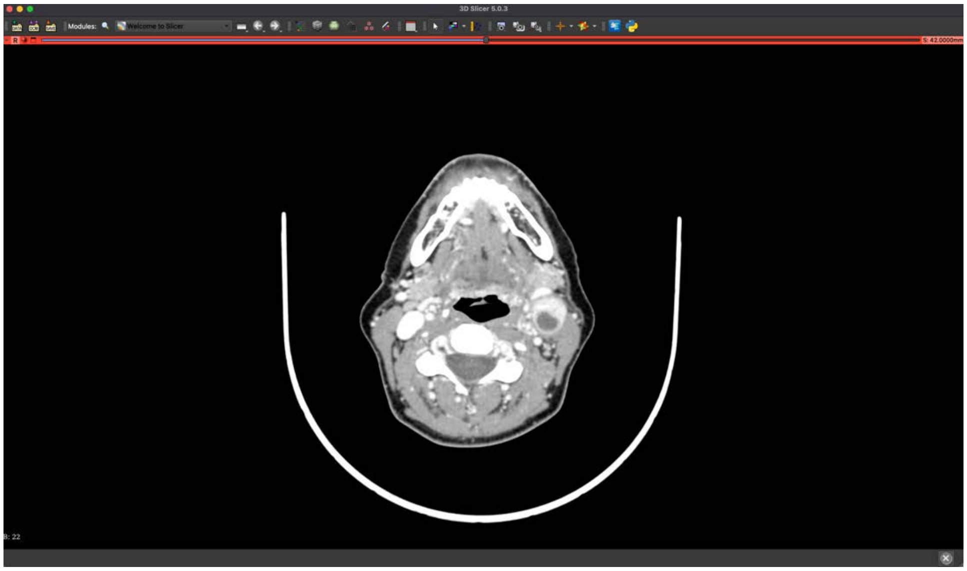

Supplementary figure 1. Example of CT scan in 3D Slicer with (top) and without (bottom) ENE presence as seen by observers. Observers could scroll through the scan remotely, change planes between axial, sagittal, or coronal, and change the window level and width.

medRxiv preprint doi: https://doi.org/10.1101/2023.02.25.23286432; this version posted June 8, 2024. The copyright holder for this preprint
(which was not certified by peer review) is the author/funder, who has granted medRxiv a license to display the preprint in perpetuity.
It is made available under a CC-BY 4.0 International license .

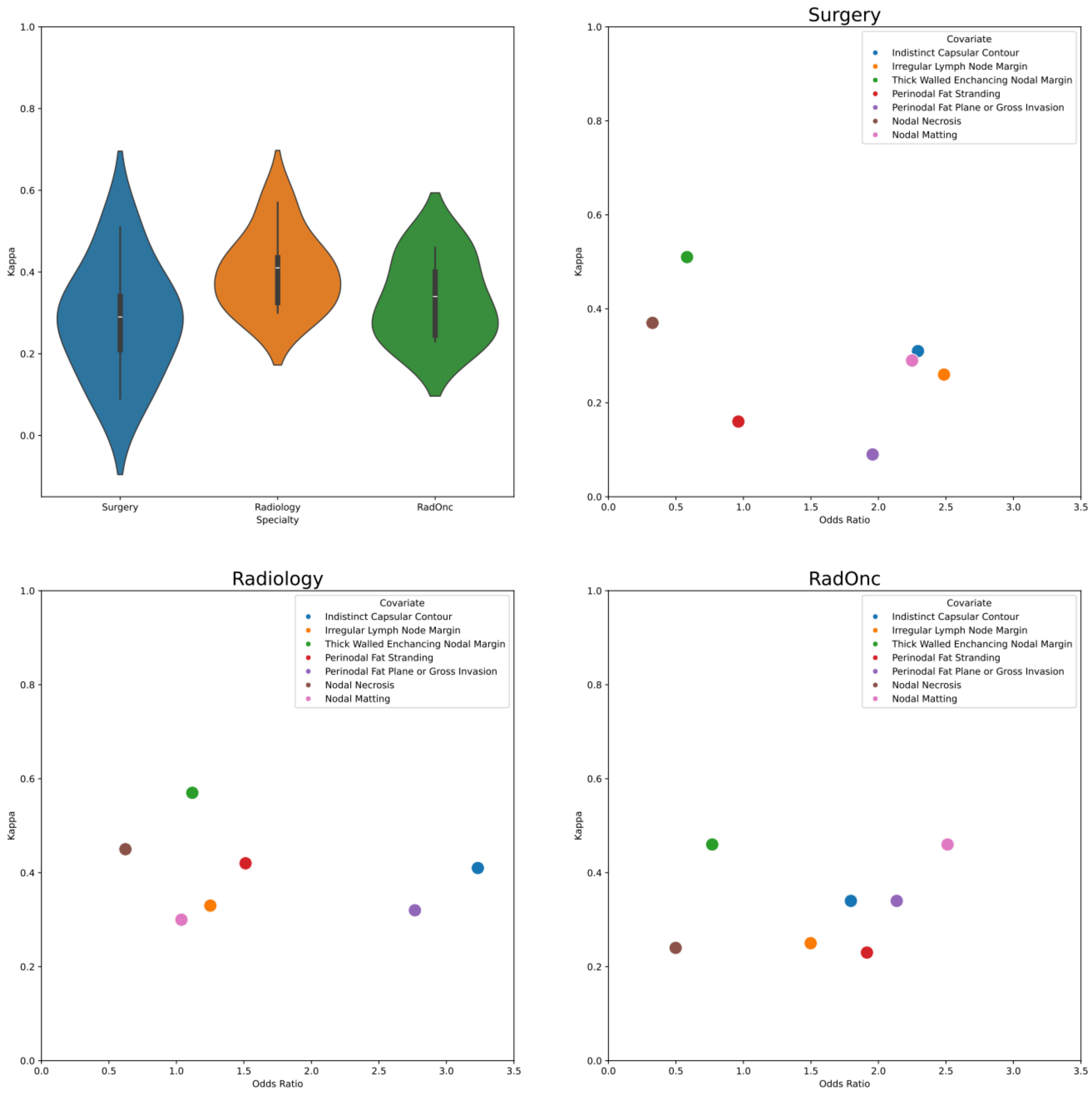

Supplementary figure 2. Fleiss' Kappa for the seven radiographic features for each specialty. Higher values represent greater agreement in the evaluation of presence or absence for each feature. Subplots show agreement versus odds ratio in correctly determining ENE for each feature stratified by clinician specialty. The top right corner of the subplots represents features with high agreement and high predictive value.

medRxiv preprint doi: https://doi.org/10.1101/2023.02.25.23286432; this version posted June 8, 2024. The copyright holder for this preprint (which was not certified by peer review) is the author/funder, who has granted medRxiv a license to display the preprint in perpetuity.
It is made available under a CC-BY 4.0 International license .

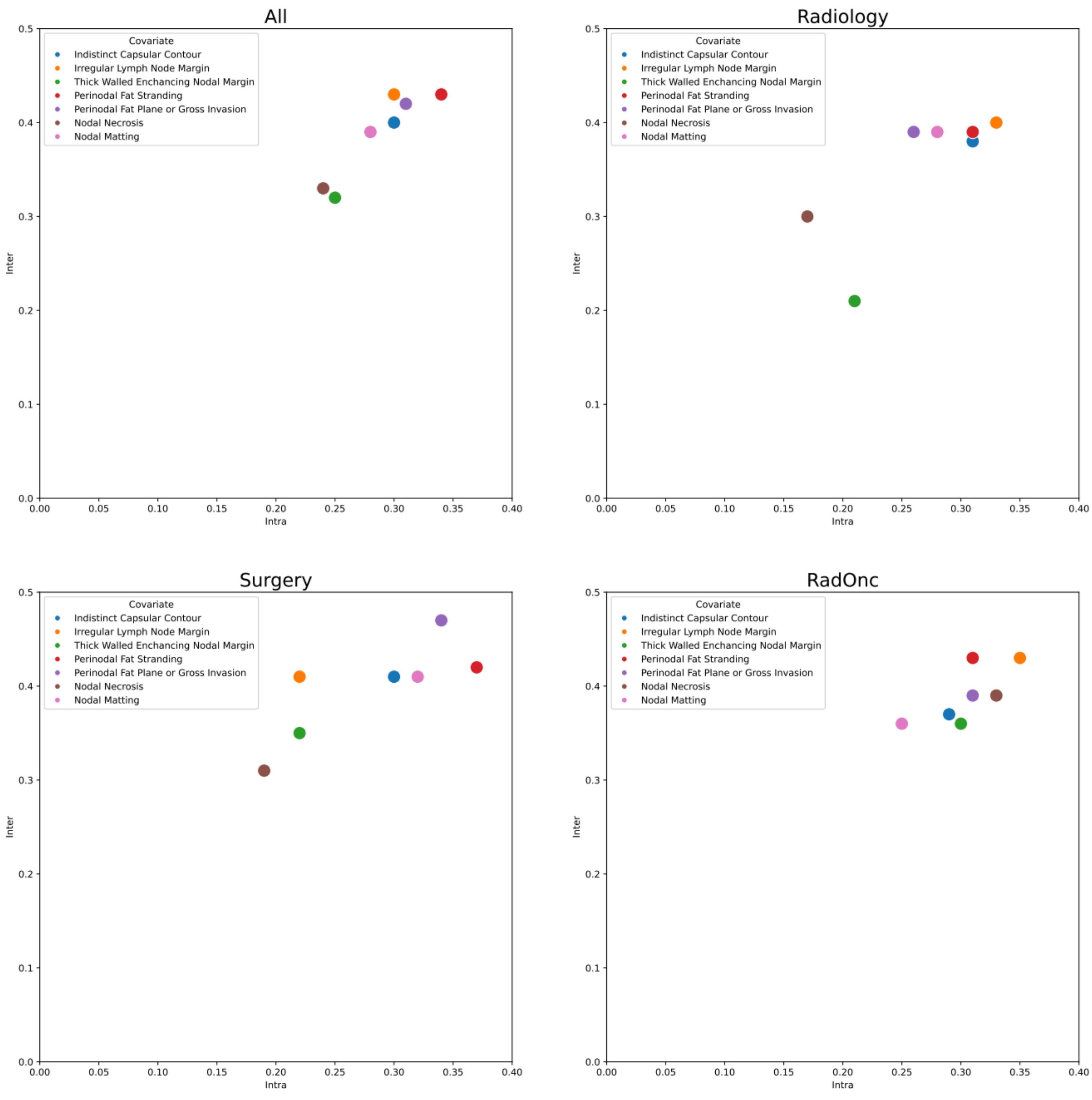

Supplementary figure 3. Interobserver vs. intraobserver variability plots as measured with the standard error of measurement. Each colored dot corresponds to a radiographic criterion. Results are presented for all observers and stratified by clinician specialty. Values in the bottom left corner represent features with low interobserver variability and low intraobserver variability, so would be preferred.
